# Aging effects on airflow dynamics and lung function in human bronchioles

**DOI:** 10.1371/journal.pone.0183654

**Published:** 2017-08-28

**Authors:** JongWon Kim, Rebecca L. Heise, Angela M. Reynolds, Ramana M. Pidaparti

**Affiliations:** 1 College of Engineering, University of Georgia, Athens, Georgia, United States of America; 2 Department of Biomedical Engineering, Virginia Commonwealth University, Richmond, Virginia, United States of America; 3 The VCU Johnson Center, Virginia Commonwealth University Medical Center, Richmond, Virginia, United States of America; 4 Department of Mathematics & Applied Mathematics, Virginia Commonwealth University, Richmond, Virginia, United States of America; Technion Israel Institute of Technology, ISRAEL

## Abstract

**Background and objective:**

The mortality rate for patients requiring mechanical ventilation is about 35% and this rate increases to about 53% for the elderly. In general, with increasing age, the dynamic lung function and respiratory mechanics are compromised, and several experiments are being conducted to estimate these changes and understand the underlying mechanisms to better treat elderly patients.

**Materials and methods:**

Human tracheobronchial (G1 ~ G9), bronchioles (G10 ~ G22) and alveolar sacs (G23) geometric models were developed based on reported anatomical dimensions for a 50 and an 80-year-old subject. The aged model was developed by altering the geometry and material properties of the model developed for the 50-year-old. Computational simulations using coupled fluid-solid analysis were performed for geometric models of bronchioles and alveolar sacs under mechanical ventilation to estimate the airflow and lung function characteristics.

**Findings:**

The airway mechanical characteristics decreased with aging, specifically a 38% pressure drop was observed for the 80-year-old as compared to the 50-year-old. The shear stress on airway walls increased with aging and the highest shear stress was observed in the 80-year-old during inhalation. A 50% increase in peak strain was observed for the 80-year-old as compared to the 50-year-old during exhalation. The simulation results indicate that there is a 41% increase in lung compliance and a 35%-50% change in airway mechanical characteristics for the 80-year-old in comparison to the 50-year-old. Overall, the airway mechanical characteristics as well as lung function are compromised due to aging.

**Conclusion:**

Our study demonstrates and quantifies the effects of aging on the airflow dynamics and lung capacity. These changes in the aging lung are important considerations for mechanical ventilation parameters in elderly patients. Realistic geometry and material properties need to be included in the computational models in future studies.

## Introduction

Many patients with obstructive lung diseases such as Chronic Obstructive Pulmonary Disease (COPD), asthma, pneumonia, and Acute Respiratory Distress Syndrome (ARDS) need mechanical ventilation in order to support their breathing and restore respiratory function. However, mechanical ventilation can cause further lung injury. The mortality rate for patients on mechanical ventilation is approximately 35% and this rate surges up to about 53% for the elderly [1]. Also, with increasing age, the severity of ventilator-induced lung injury (VILI) increases [2]. In general, with increasing age, the dynamic lung function and respiratory mechanics are compromised. In this study, several simulations are carried out to estimate these changes and understand the underlying mechanisms to better treat elderly patients.

Various morphological and tissue parameters of human lungs change due to aging. For example, there is an approximate 10% reduction in the diameters of the lower bronchioles (G12~) between 50 and 80 years of age [3]. In addition, the alveolar sacs increase in size with age [4]. In general, there is a steady 1% decrease per year in respiratory mechanics and lung function after about 30 years of age [5, 6]. Lung tissue becomes approximately 7% stiffer from 50 to 80 years of age [5]. Lung compliance represents the elastic property of the lung and is a volumetric quantity that depends on the size of the lung. Compliance is defined in the relationship with volume and pressure, and is essentially the ability of the lung tissue to “absorb” the same applied force, which generally results from a change in pressure. In general, compliance increases with aging [7]. Compliance is an extrinsic parameter, which increases if alveolar sacs increase in size. Lungs with low compliance are stiff lungs and will require much greater pressure to reach a given volume. A stiff lung would need a greater than average change in pleural pressure to change the volume of the lungs, and breathing becomes more difficult as a result. Hence, in this study, we investigated lung compliance to estimate elastic properties in aging, and measured volume, pressure and strains to assess compliance and stiffness due to the aging effect. Computational models were used to further assist in understanding age related changes in airflow dynamics and respiratory mechanics.

Several experiments were previously conducted on mice [8–10] in order to evaluate the underlying mechanisms in aging, For example, Jonathan et al [9] mechanically ventilated mice at 10 ml/kg tidal volume for about 3 minutes prior to initiating mechanical perturbations to investigate respiratory system mechanics and lung morphology across a more complete spectrum of age groups, 2-, 6-, 18-, 24-, and 30-month-old mice. This experiment showed the aging related decrease in respiratory system resistance and increase in lung compliance between 2-month-old and 24-month-old mice. Recently, Herbert et al [10] mechanically ventilated 2-month-old and 20-month-old male mice and established that older mice had increased lung compliance and were more prone to ventilator-induced edema and mortality.

Experimental studies related to lung compliance in human subjects have also been reported [4, 11]. For example, Bozanich *et al*. [12], Sly *et al*. [13] and Kewu [8] observed that decreases in elastin containing fibers are associated with increases in lung compliance. The bulk modulus of the lung is a factor that indicates stiffness and properties of the lung tissue at a given volume. A recent study reported that bulk modulus increases linearly with age [5, 14]. Other experimental studies have reported that the lung elastic recoil (inversely related to the lung compliance) decreases with age [4, 15, 16]. Additionally, an increase in alveolar surface area [17, 18] is also associated with aging. In order to understand the mechanisms of aging, the properties of the small bronchioles and alveoli must be examined as the aging effects are more pronounced in small bronchioles and alveoli. Both the material properties of bronchioles and alveoli as well as geometrical parameters (diameter and thickness) change due to aging. Hence, we assumed these geometric and material properties as sensitivity factors for aging in this study. An aged model (80-year-old) was developed by altering the geometry and material properties of the 50-year old model. Computational simulations using coupled fluid-solid analysis were performed for geometric models of bronchioles and alveolar sacs under mechanical ventilation to estimate the airflow and lung function characteristics.

In addition to experimental studies, several computational studies using rat models [19–22] have been completed of lung airways and alveolar sacs in normal lungs and emphysematic subjects to assess lung function. Oakes et al. [23] used MRI-based data. Moreover, human aged lungs were studied by [24, 25]. Xia et al. [26] used a fluid-structure interaction method to simulate the flow and airway wall stiffness.

The majority of the previous computational studies were performed on normal airways and diseased airways. There are some experimental studies [3–5] dealing with aging effects in terms of elastic properties of lungs. However, there are no computational studies investigating aging effects on lung mechanical parameters. Hence, there is a need to investigate the aging effects on respiratory mechanics and lung function through computational studies. This study investigates aging effects through computational simulations.

In this study, aging effects were addressed through computational simulations of morphological and tissue property changes to investigate the lung function characteristics. The simulation model consists of two geometries, bronchioles from generation 10 to generation 22 and alveolar sacs (G23). An integrated model was constructed by combining the bronchiole regions and alveolar sacs of the lung and the lung mechanics were investigated under mechanical ventilation. The results of respiratory mechanics parameters as well as lung function obtained from the computations are discussed.

## Materials and methods

We split our model into two regions, G1 ~ G9, and G10 ~ G23 (where aging effect was considered). The reason for doing so was to address the computer memory load required to numerically calculate data for the whole region from G1 ~ G23. Thus, first we collected flow information at the outlet of the G1 ~ G9 model, which is based on dimensions of anatomical cases [26] and then applied the information to the inlet for G10 ~ G23. The G10 ~ G23 model was separately generated according to population-averaged lung data [24, 25] and it was connected to the G1 ~ G9 model with scaling. As the aging effect is more pronounced at distal generations (G10 ~ G23), we therefore focused more on G10 ~ G23 in this study.

Four models were considered in this study, one representing normal (50-year-old) and three aging (60-, 70-, and 80-year-old) airway models with realistic geometric dimensions based on the literature review [3, 27]. These models along with airway tissue material properties, and input and boundary conditions are presented in this study.

### Geometric models

**Fig 1:**
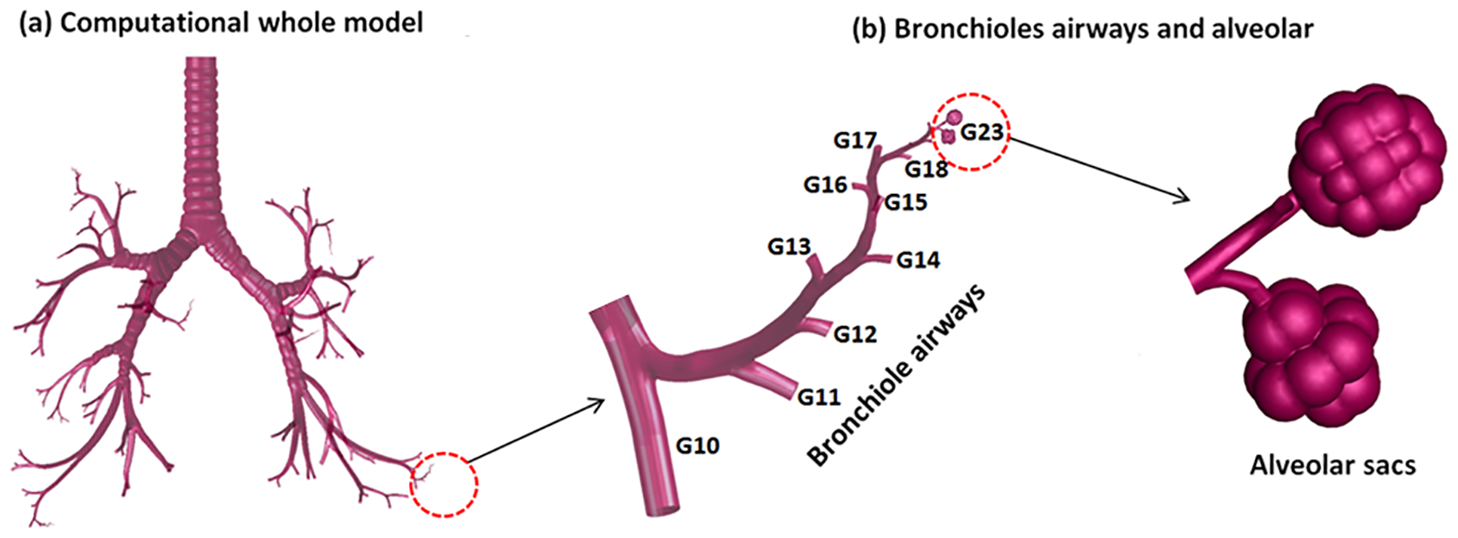
Human tracheobronchial airway model (a), and bronchioles and alveolar sacs. G10 ~ G23 were region of interest where aging effects are considered in this study.

Fig 1 shows the tracheobronchial (G1 ~ G22) as well as alveolar sacs (G23) models developed in this study, and the aging effect was considered for G10 ~ G23. Additional details for the G10 ~ G23 model are also presented in Tables 1 and 2. Initially, the computational surface model of the respiratory tract for a tracheobronchial (TB) whole lung model (G1 ~ G9) shown in Fig 1a was developed based on anatomical case dimensions reported by Yeh and Schum [28] and scaled to a functional residual capacity (FRC) of 3.5 L, which is consistent with a 50-year-old male [29]. The three major features of this physiologically realistic TB model (G1 ~ G9) are the right-left asymmetry, the cartilage rings, and the non-planar bifurcating branches. There are two lobes (upper and lower) in the right lung. Similarly, ventilation to the right and left lungs is also asymmetric. C-shaped cartilage rings were kept in the TB model through the trachea to the bifurcation G4, which prevent these airways from collapsing during absence of air [30]. Surface properties of the bifurcations such as the carina ridge were taken from the measurements of Horsfield et al. [31] and Hammersley and Olsen [32]. The bifurcation units were rotated out of plane to approximate the gravity angles specified by Yeh and Schum [28]. The branch diameters, lengths, and bifurcation angles of each generation are consistent with those reported by Heistracher and Hofmann [33], with only slight modifications that were required to generate smooth asymmetrical bifurcations. Based on the diameter and length dimensions reported in previous studies [28, 33], the first generation of airway was created, and the right main stem bronchus was regenerated that forms a 20 to 30 degree angle with the vertical axis [33]. For the left main stem bronchus, a 45 to 55 degree angle was formed with the vertical axis. For the lobar bronchi, we divided the right main stem bronchi into three lobar bronchi (upper, middle and lower), and the left main stem bronchi into two lobar bronchi (upper and lower), and we created segmental bronchi, bronchioles, and alveolar sacs that form a 20 to 40 degree angle with the vertical axis.

**Table 1:** Geometrical changes in generation diameter (D) and length (L) with age adopted in this study.

Generation number	Diameter (D) and centerline length of each generation (L) (mm)							
	50-year-old		60-year-old		70-year-old		80-year-old	
	D	L	D	L	D	L	D	L
**10**	1.85	7.31	1.67	6.579	1.48	5.848	1.30	5.117
**11**	0.99	5.35	0.89	4.815	0.79	4.28	0.69	3.745
**12**	0.79	4.94	0.71	4.446	0.63	3.952	0.55	3.458
**13**	0.62	3.36	0.56	3.024	0.50	2.688	0.43	2.352
**14**	0.49	2.76	0.44	2.484	0.39	2.208	0.34	1.932
**15**	0.38	1.76	0.33	1.584	0.30	1.408	0.26	1.232
**16**	0.37	1.55	0.32	1.395	0.29	1.24	0.25	1.085
**17**	0.36	1.15	0.34	1.035	0.30	0.92	0.27	0.805
**18**	0.28	1.07	0.25	0.963	0.22	0.856	0.20	0.749
**19**	0.25	0.96	0.23	0.864	0.20	0.768	0.18	0.672
**20**	0.22	0.84	0.20	0.756	0.18	0.672	0.15	0.588
**21**	0.18	0.75	0.16	0.675	0.14	0.6	0.13	0.525
**22**	0.16	0.63	0.14	0.567	0.13	0.504	0.11	0.441
**23**	0.08	0.45	0.07	0.405	0.06	0.36	0.05	0.315

Remarks: The diameter and centerline length of 50-year-old in this study was employed from Weibel’s study [27] of branching airways and diameters of other age groups was calculated by ratio from experimental measurements [3, 24].

**Table 2:** Tidal volume and airway parameter used in the simulation model.

		50-year-old	60-year-old	70-year-old	80-year-old
**Airway space volume (c*m*^*3*^)**	Trachea	67.455	67.455	67.455	67.455
	Bronchioles	4.77	3.48	2.53	1.78
	Alveolar sacs	0.00133	0.00291	0.00544	0.00911
**Surface area (c*m*^*3*^)**	Trachea	259	259	259	259
	Bronchioles	33.2	27.2	21.9	17.3
	Alveolar sacs	0.1268	0.2116	0.3136	0.4422
**Tidal volume (mL)**		420	420	420	420

Remarks: Airways of each age are generated based on Table 1 using Gambit and Ansys. The value of tidal volume was applied from [8–10]. Tidal volume was applied in all aged model from the data in G1 ~ G9 simulation.

This TB model only included trachea to generation 9. In order to complete the whole region (G1 ~ G23), we generated bronchiole regions with specific diameters from [27] as well as dimensions for alveolar sacs [34] according to population-averaged lung data using Gambit and ICEM CFD(Fig 1b). Finally, separate bronchiole regions and alveolar sacs of different sizes were attached to the TB model (G1 ~ G9) in order to obtain the model for the whole region of the lung (Fig 1a). For the aging model, there was no significant difference in the mean value for the mean segmental bronchus (G2 ~ G9) diameter with age [3]. However, considerably greater variation was found in the measurement of the mean bronchioles (G10 ~) diameter that ranged from 0.5 to 1.048 mm with age [3, 27]. A review of the literature suggested that there are age-related diameter reductions (10% reduction for a 60-year-old, 20% reduction for a 70-year-old, and 30% reduction for an 80-year-old) in the bronchioles airways as shown in Table 1. These reductions were regenerated at generations G10 ~ G22 with diameters from [3, 27] by using Gambit and ICEM CFD to account for aging effects in the simulated geometric models. The alveolar (G23) sizes also were regenerated using previous experimental data [34], and the alveolar space and surface area measurements are presented in Table 2. Two alveoli were employed for simulation and the bigger one was analyzed in this study. A single alveoli is best approximated as a 5/6 spherical cap [35] and, hence, we employed such a spherical model mounted on a respiratory bronchiole consisting of a circular airway, as shown in Fig 1. We assumed the lumen thickness was the same for the normal case generation we focused on in this study (G10 ~). A previous study by M. Montaudon et al. [36] on assessment of bronchial wall thickness reported no significant effect on thickness in the lower generation. The aging models reflected a reduction of airway (lumen) diameter due to increasing thickness of the airway wall (increasing of thickness of collagen and membranes in the lungs). The thickness dimensions for each age group were based on a review of the literature [5, 36–38], and the parameters used in this study are presented in Table 3.

**Table 3:** Material properties and thickness of airway wall (tissue) with age used in the model.

	Bronchioles		Alveolar sac	
**Age**	Initial shear modulus (Pa)	Wall thickness (mm)	Initial shear modulus (Pa)	Wall thickness (mm)
**50**	112000	0.008	35714	0.004
**60**	115920	0.0088	36964	0.0044
**70**	119840	0.0096	38214	0.0048
**80**	123760	0.0104	39464	0.0052

Remarks: Material properties and thickness of airway wall used in this study was employed by experimental measurement data [3, 38] for 50-year-old case. The parameters of other age groups were calculated from experimental measurement data [5, 36–38].

### Computational models

The computational models were generated from the geometries given in Fig 1 and Tables 1 and 2 using several software programs, Gambit, ICEM CFD, and DesignModuler. Computational simulations, specifically FSI (Fluid-Solid Interaction) studies were carried out on ANSYS Workbench using ANSYS Mechanical (Version 15.0) for the structural analysis, and ANSYS Fluent for computational fluid dynamics (CFD) analysis. The individual models (fluid and solid) were coupled using a fluid-structure interaction (FSI) algorithm.

Airflow in the airway and at the airway wall was assumed to be laminar and incompressible. Therefore the 3-dimensional incompressible laminar Navier-Stokes and continuity equations in a 3D mesh with a control volume approximation were solved numerically to obtain the velocity field within the airways.

∇⋅u=0 (1)

$$\rho \left(\frac{\partial u}{\partial t}+u\cdot \nabla u\right)=-\nabla p+\mu \nabla^{2}u$$ (2)

where *u* is the velocity field of the fluid, *ρ* is the density of fluid, *p* is the pressure, and *μ* is the dynamic viscosity. ‘u’ in the convection term of momentum equation is the fluid velocity obtained as a result of numerical computation of fluid mesh. Fluid mesh and solid mesh were coupled in multi-physics fields with moving boundaries and interfaces. The fluid boundary changes with time, and the grid velocity becomes a convective mesh velocity as per arbitrary Lagrangian Eulerian methods. The continuity equation represents the conservation of mass (Eq (1)), and the Navier-Stokes equations represent the conservation of momentum (Eq (2)), and were solved numerically on a moving grid using a commercial finite-volume method.

The fluid model equations were solved first to obtain results for fluid pressure and shear stresses, which were then applied to the solid model. The governing equations for movement of the airways during mechanical ventilation are time-dependent structural equations that are described by examining the forces acting on airways in a fluid, and are given as

$$\rho \frac{Du}{Dt}=\nabla \cdot \sigma +f$$

σ=C⋅ε

where σ is the Cauchy stress tensor, and *f* accounts for other external forces present and ε is the infinitesimal strain tensor (also called Cauchy strain tensor). The strain and stress tensor can be represented as

$$\varepsilon =\left(\begin{array}{ccc} \varepsilon_{xx} & \varepsilon_{xy} & \varepsilon_{xz}\\ \varepsilon_{yx} & \varepsilon_{yy} & \varepsilon_{yz}\\ \varepsilon_{zx} & \varepsilon_{zy} & \varepsilon_{zz} \end{array}\right),\;\sigma =\left(\begin{array}{ccc} \sigma_{xx} & \tau_{xy} & \tau_{xz}\\ \tau_{yx} & \sigma_{yy} & \tau_{yz}\\ \tau_{zx} & \tau_{zy} & \sigma_{zz} \end{array}\right)$$

where τ is shear stress.

The airway tissue was assumed to be a hyper-elastic material in the non-linear material model. Among various available models, the popular Neo-Hookean model was used for the airway tissue [39]. The hyper-elastic Neo-Hookean material model does not predict an increase in modulus at large strains and is typically accurate only for strains less than 20%. Hence, an infinitesimal strain tensor was used, and Cauchy strain and stress tensor were employed in this study. The strain energy density function for an incompressible Neo-Hookean material in a 3D model is

$$W=C_{1}\cdot (I_{1}-3)$$

where C_1_ is a material constant, and I_1_ is the first invariant of the right Cauchy-Green deformation tensor, i.e.,

$$I_{1}=\lambda_{1}^{2}+\lambda_{2}^{2}+\lambda_{3}^{2}$$

where λ_i_ are the principal stretches. The lung tissue is modeled as a hyperelastic material represented as a Neo-Hookean model involving a single parameter. This representation provides a mathematically reliable constitutive model for the deformation behavior of lung tissue at bronchioles and alveolar sacs. C1 was used as the initial shear modulus in the hyper-elastic Neo-Hookean model in the ANSYS program. Initial shear modulus values at each age for the bronchiole (G10 ~ G22) and alveolar regions (G23) were generated based on the literature review [5, 38, 40–42] and are presented in Table 3. Displacements were solved in the solid model equations, and were then applied to the fluid model. The fluid model equations were resolved using the structural displacements at the boundaries. The process iterated until a converged solution was found for each time step.

### Inputs and boundary conditions

The inlet boundary condition for the fluid domain was a waveform during mechanical ventilation. The flow was imposed at an inlet at each iteration. In normal clinical practice, constant flow during inhalation and exponential decay during exhalation has become established as the standard practice for ventilator control [43]. Standard clinical ventilator practice also indicates that there is less volume in the lung during exhalation than inhalation [44]. In this study, the respiratory rate (I:E ratio) was reduced to allow more time for exhalation and reduce breathing stacking. We generated an user-defined function based on the standard clinical practice for ventilation and the exhalation time was adjusted under mechanical ventilation, and the solution was independent of the number of breathing cycles in the simulation.

The airflow rate during mechanical ventilation is assumed to be a constant 60 L/min for inhalation [31, 32] in the G1 ~ G9 simulation. A peak flow rate of 60 L/min may be considered to be sufficient although higher volume flow rates may be needed for some patients. Ventilated waveform was imposed at the bronchiole inlet (G10) based on a previous study [43]. The total breath cycle was 2 seconds with an inhalation period of 0.4 seconds and exhalation period of 1.6 seconds. The tidal volume is 420 mL for all models. Under mechanical ventilation, a positive constant flow rate during inhalation (0–0.4 second) and negative exponential flow rate waveform during exhalation (0.4–2 second) were considered. The calculated velocities of airflow of transient breathing waveforms are given as:

$$u(t)=\frac{Q}{F(A)\cdot 2^{n-1}}$$ - Inhalation

$$u(t)=\frac{-Q\cdot e^{\frac{t}{0.4}}}{F(A)\cdot 2^{n-1}}$$ - Exhalation

where *Q* is flow rate (tidal volume/inhalation) obtained from G1 ~ G9 simulation results, *F(A)* is total face area obtained by calculating the magnitude of each face’s area units defined in software Fluent 15, and *n* is the generation number.

In the realistic lung, alveolar sacs are filled with air initially before air is inhaled. Therefore, the air was inhaled from the mechanical ventilation in the simulation. To simulate the initial air filled condition, u, v, w, and p were set to non-zero (but very small) values at t = 0. In this condition, initial Reynolds number of flow would be relatively small, but flow at t = 0 would not be zero. The initial air was filled by adjusting tidal volume through the user-defined function in the ANSYS program. The user-defined function is given as a data file along with the manuscript.

Pressure boundary waveform was applied to all outlets of G11 ~ G22 and alveolar sacs (G23) based on the data presented by Pedley [45], and waveforms were generated based on a previous study [43]. For modeling the airway tissue from G10 through G23 using the Neo-Hookean material model, an initial shear modulus value needs to be specified for simulation in ANSYS. This value is specified as an input to the model in ANSYS software. Initial shear modulus was applied differently on the tissue of bronchioles (G10 ~ G22) and alveolar sacs (G23) as indicated in Table 3.

### Simulations

**Fig 2:**
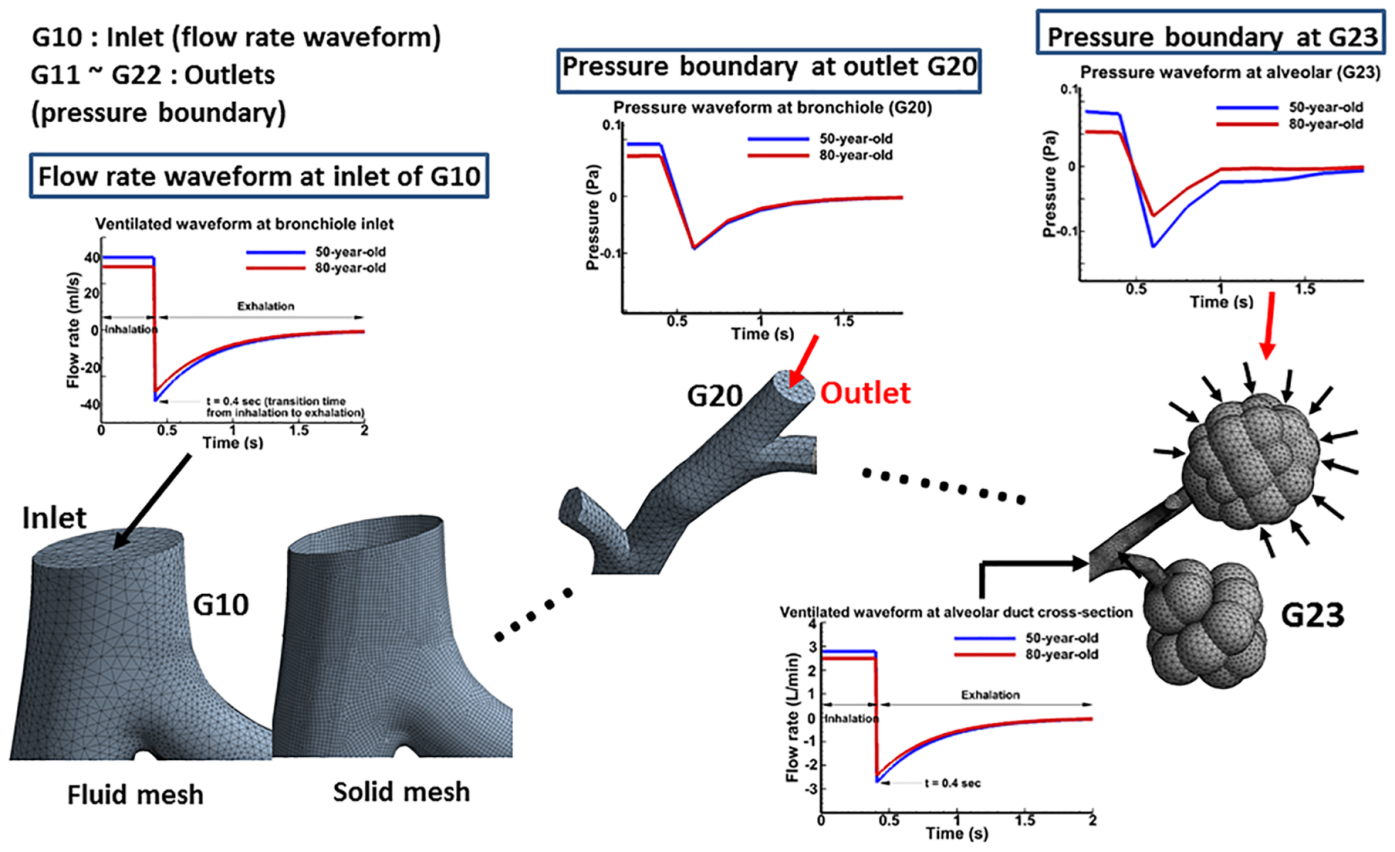
Boundary conditions for input, output, and alveolar sacs with computational mesh. Ventilated waveform of flow rate at inlet G10, pressure waveform at all outlets (this figure shows pressure boundary condition at the outlet G20), and pressure waveform at the alveolar sac, were considered as boundary conditions for the 50-year-old and 80-year-old lungs. For the computational mesh, solid and fluid mesh elements of bronchioles were approximately 229,791 and 832,960; and for alveolar sacs they were approximately 82,530 and 201,432 respectively. The solid part was simulated on a single tissue layer.

Simulations were performed with normal (50-year-old) and aged (80-year-old) models under mechanical ventilation waveform to estimate lung function characteristics. Fig 2 shows the computational meshes for both the fluid and solid domains of the geometric models. The fluid domain of the model was comprised of 1,034,392 tetrahedral elements with 394,492 nodes, and the solid domain had 312,321 tetrahedral elements with 219,293 nodes. 119327 elements were used in the thickness direction for the solid. For the fluid structure interaction, a loose coupling method was used. The finite element mesh developed in this study is partitioned with a mesh density of 0.2 (size factor) in ANSYS software.

In a loose coupling scheme, governing equations for incompressible were solved only once per time step leading to a solution at each time [46]. Added mass effect has been observed in loose coupling of fluid and structural parts in the context of incompressible flow and slender structures. In fluid-structure interaction, on short time scales, the fluid appears as an added mass to the structural operator, and the stability and convergence properties of the sub-iteration process depend significantly on the ratio of this apparent added mass to the actual structural mass. At increasingly small time intervals, the added mass of an incompressible flow approaches a constant.

For the fluid part, the Eulerian method was used and the structure was treated as an embedded moving boundary. A FEM solver for the structure was employed based on the Lagrangian framework. Both fluid and structure meshes are independent and at the fluid-structure interface (FSI), the meshes conform to each other. The interface boundary locations and velocities were dictated by the Lagrangian structure. This information was used by the fluid solver as the interface conditions at each time step, and fed back as the fluid pressures on the structural interface as exterior forces for the structure solver. In the ANSYS program, all FSI boundaries will deform with conforming meshes involving three fields that describe the fluid dynamics, structural dynamics and mesh movement, respectively. When the volume mesh is implemented, the meshes at FSI boundary will conform to each other. An iterative process involving coupled fluid-structural computations was carried out to ensure that the interface conditions of both the displacement and the force are satisfied at a given time before moving to the next time step. This is a standard procedure in ANSYS software and we followed this in the simulation.

No slip boundary consideration was assumed at the fluid-solid interface, implying that there is no relative velocity between fluid and solid mesh boundaries. The fixed-point iteration method was employed in the fluid-solid coupled simulation with 4000 iterations. To verify the accuracy of the solution, the interface domain between fluid and solid was discretized with three different meshes with increasing numbers of elements. For each mesh, the von-Mises stress was calculated and it was found that a mesh with 620,000 elements reached convergence and this element size was used in the simulations.

It was found that the highest Reynolds number for the flow was 1055 at the 1^st^ generation, and it was lower for subsequent airway generations. Hence, the assumption of laminar flow was assumed in the simulation. A time step size of 0.01 (s) was used in this study. Several simulations were carried out with normal and aging models to estimate the airway mechanics and lung function parameters. Strains and stresses were obtained by post-processing the simulation results. At each time step, the pressure and volume were estimated from the converged solutions from the simulations. After that, P-V loops for normal and aged models were generated from the simulation results of pressure and volume.

## Results

### Airway mechanics characteristics

**Fig 3:**
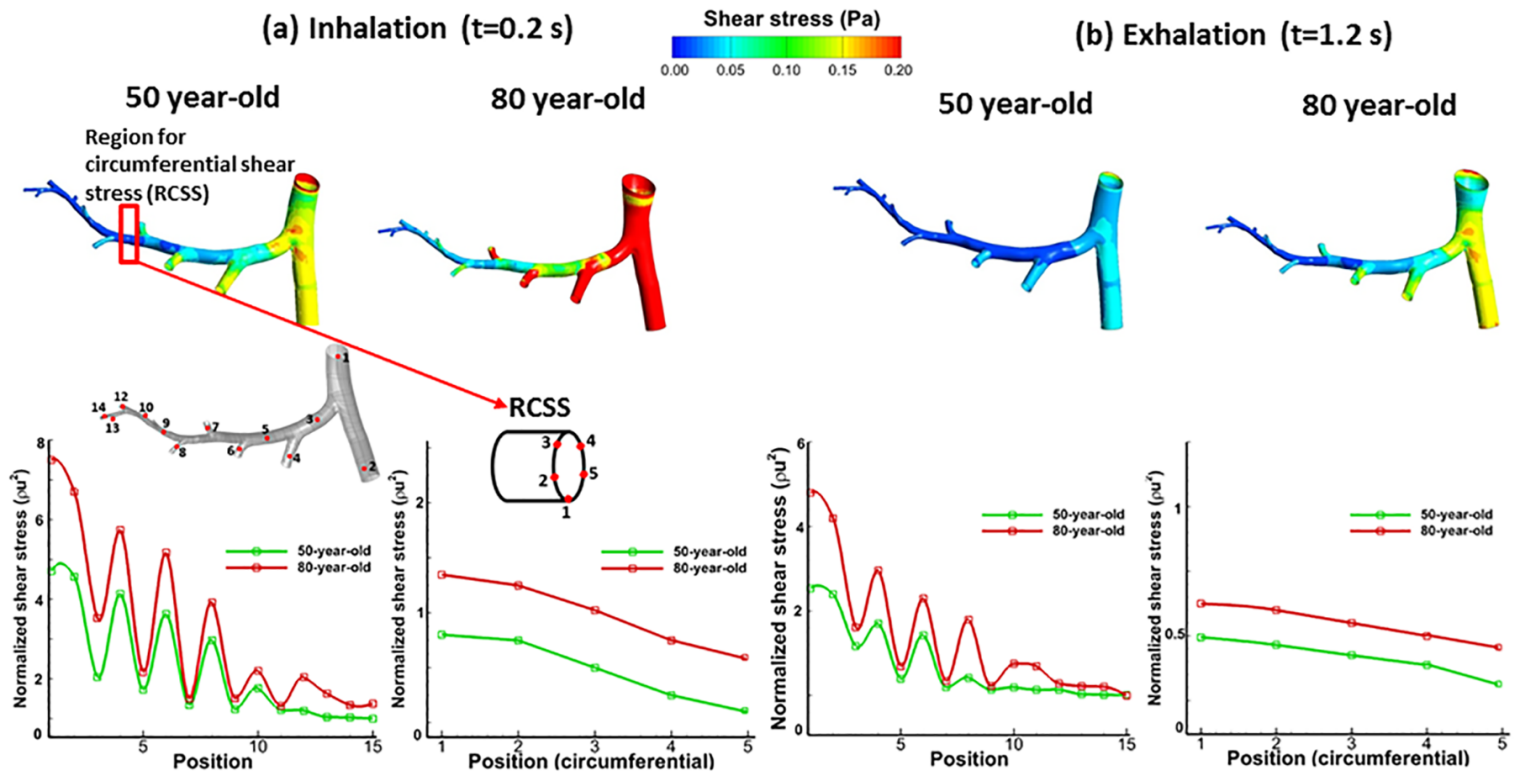
Comparison of wall shear stresses in bronchiole airways for 50-year-old and 80-year-old during mechanical ventilation; contour of shear stress, normalized shear stress by generations, and normalized circumferential shear stress.

**Fig 4:**
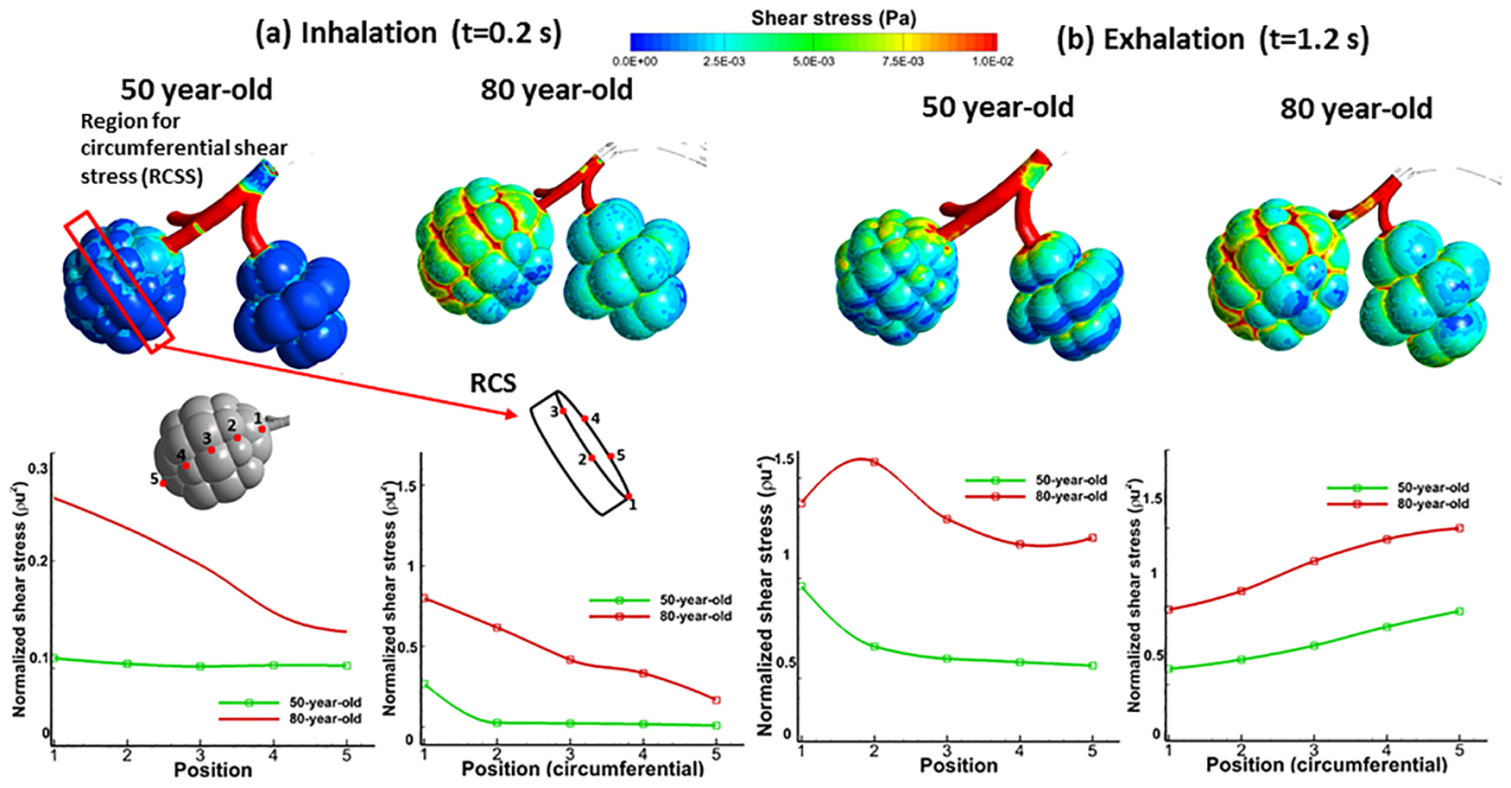
Comparison of wall shear stresses in alveolar sacs for 50-year-old and 80-year-old during mechanical ventilation; contour of shear stress, normalized shear stress by translational position, and normalized circumferential shear stress.

**Fig 5:**
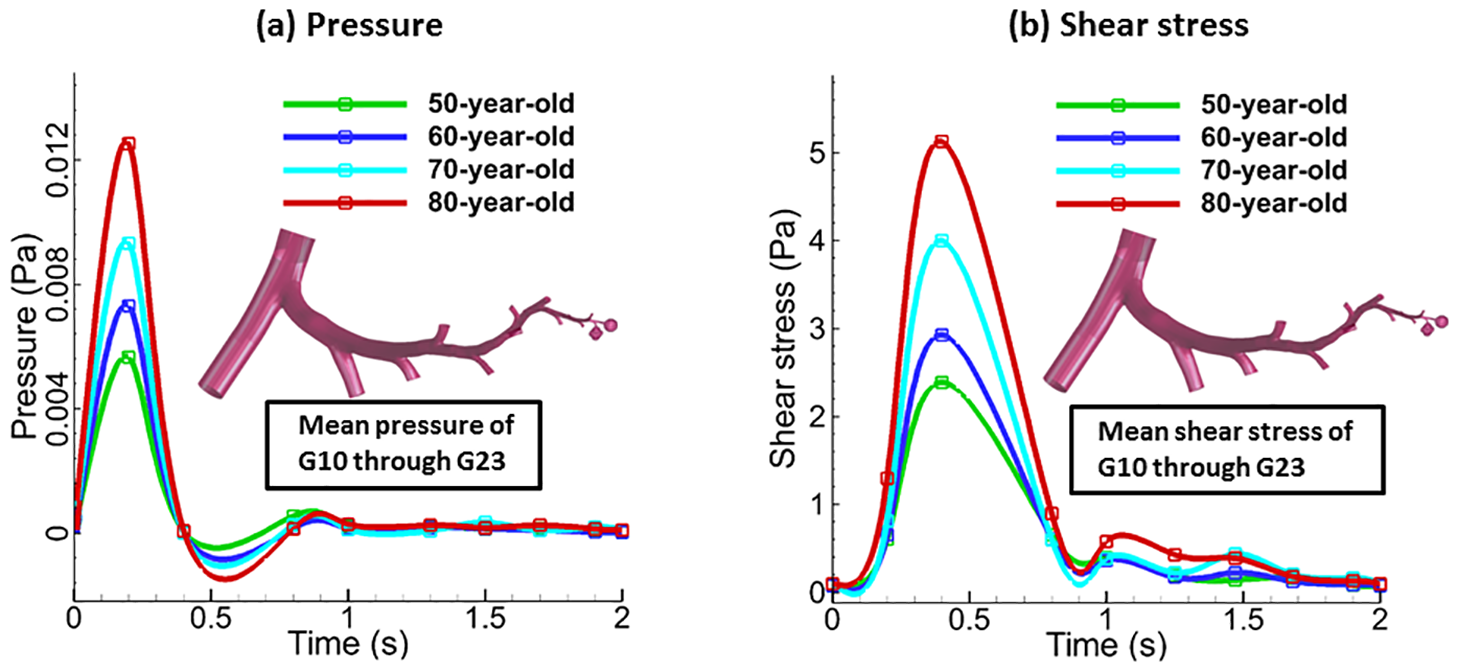
Comparison of pressure and wall shear stress by airflow for each age in the breathing cycle over the region of G10 ~ G23. Mean values were computed by simulation for each age during inhalation, 0–0.4 s and exhalation 0.4–2.0 s. The pressure and shear stress at t = 0 is very close to 0 but not 0.

**Fig 6:**
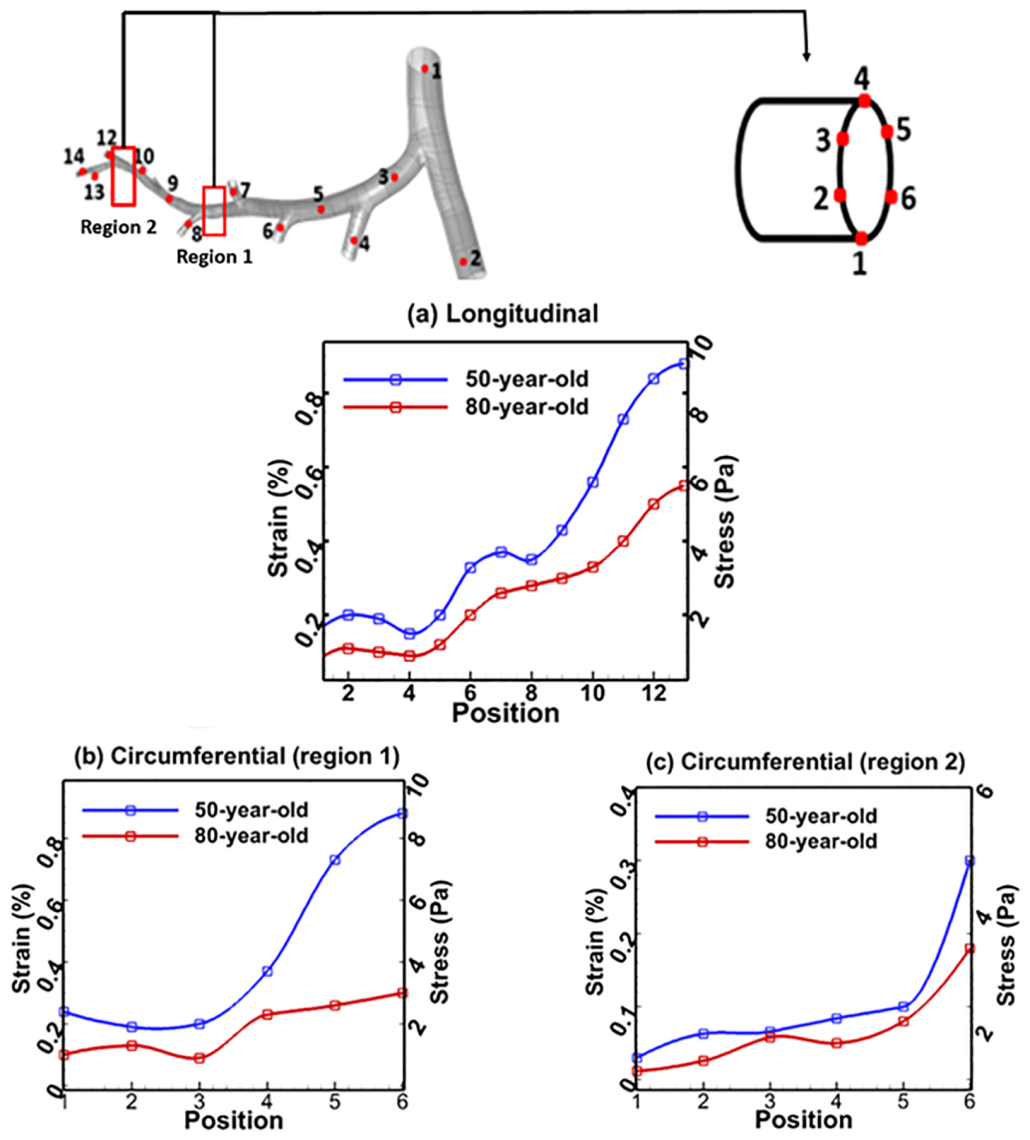
Comparison of strains in bronchiole airways between 50-year-old and 80-year-old for inhalation and exhalation at several positions; (a) longitudinal, and (b) circumferential.

**Fig 7:**
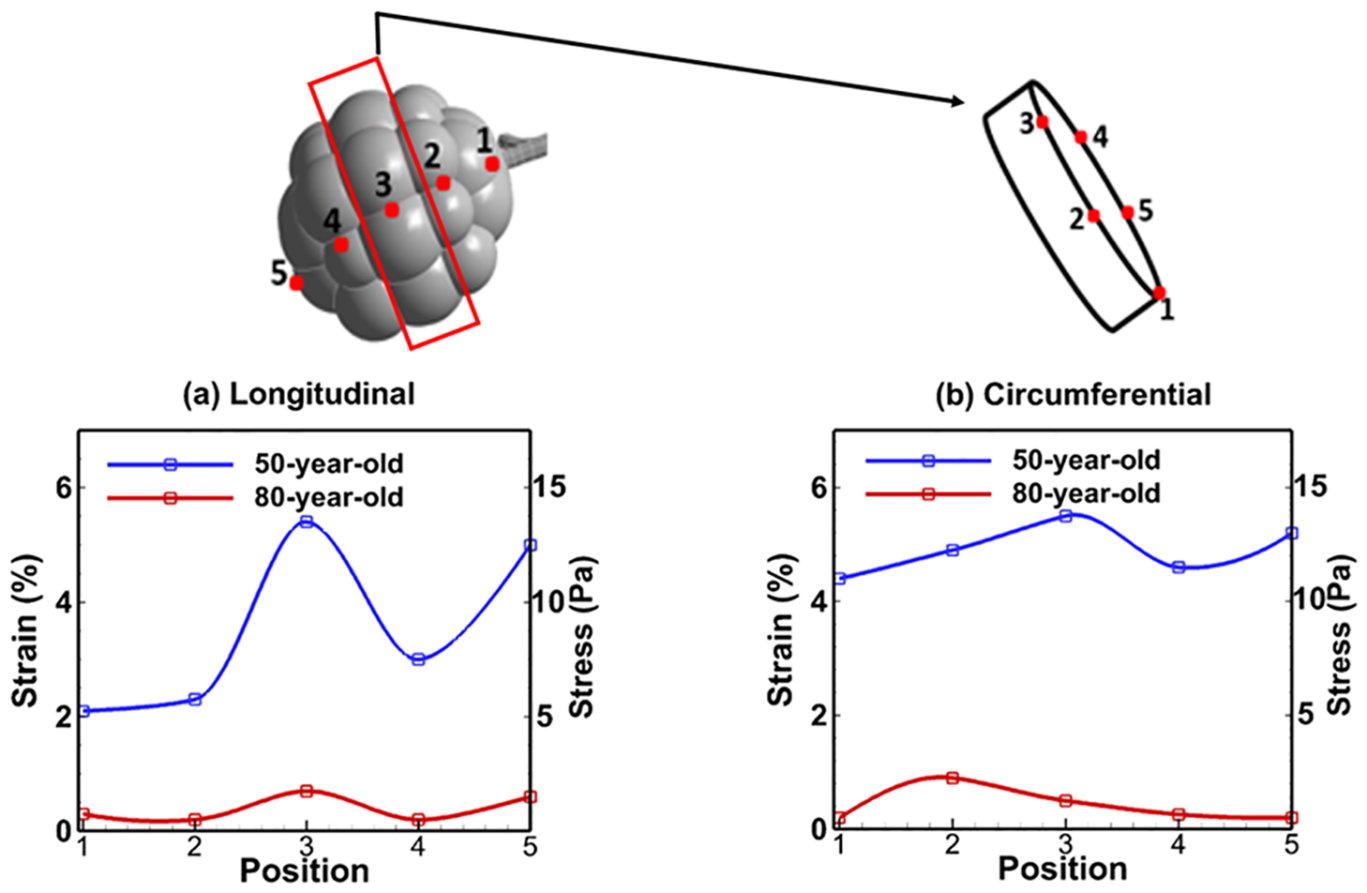
Comparison of relative change of strains in alveolar sac between 50-year-old and 80-year-old for inhalation and exhalation at several positions; (a) longitudinal, and (b) circumferential.

The airway mechanics characteristics represented by parameters (pressure, wall shear stress and the equivalent tissue strain) obtained for 50-year-old and 80-year-old models are presented in Figs 3–7. The contour plots show results at 0.2 seconds, halfway through inhalation, and at 1.2 seconds, halfway through exhalation. Shear stress is normalized with respect to the difference between the maximum and minimum shear stress. The maximum wall shear stress contours in the bronchiole airways are similar for both the 80-year old and the 50-year-old as shown in Fig 3. However, the magnitudes are different. It can be seen from Fig 3 that the overall wall shear stress is higher for the 80-year-old as compared to the 50-year-old during both inhalation and exhalation. Also, the values of shear stress decrease from G10-G22 and vary along the airway cross-section, as can be seen from Fig 3. The maximum wall shear stress increased by 56% during inhalation and by 43% during exhalation for the 80-year-old. The numerical values were measured along the alveolar sac wall from position 1 to 5 as shown in Fig 4. Similar trends were observed for the alveolar sac models of the 80-year-old case during inhalation as shown in Fig 4a. The magnitudes of wall shear stresses on alveolar sacs were much smaller than in the bronchiole airways. The maximum wall shear stress increased by 20% for the 80-year-old. In addition, the distribution of the wall shear stress varied among various sacs at different time steps during mechanical ventilation. Both the pressure and the shear stress varied with aging at different time steps during mechanical ventilation as shown in Fig 7. This Fig 7 indicates average pressure and shear stress over the region of G10 ~ G23. As the G10 ~ G20 simulation model is continuous, the results for pressure and shear stress are smooth as shown in Fig 7. For all the four age groups considered, the peak pressure was found at t = 0.2 seconds of inhalation. After that, there is a dramatic drop in the pressure during the rest of the inhalation. A 38% pressure drop was observed for the 80-year-old as compared to the 50-year-old as shown in Fig 7a. Similarly, the shear stress on airway walls also increased with aging and the highest shear stress was again observed in the 80-year-old at t = 0.4 seconds as shown in Fig 7b. Both the air pressure and wall shear stress results clearly demonstrate the effect of aging on airway mechanical characteristics. A summary of these results is presented in Tables 4 and 5.

**Table 4:** Quantification of airway mechanical parameters with age and comparison to experimental data.

			50-year-old	80-year-old	Difference (%)
**Max. Pressure (Pa)**	Inhalation	Bronchioles	0.01	0.03	33
		Alveolar sacs	0.00032	0.00012	37.5
	Exhalation	Bronchioles	0.0013	0.002	65
		Alveolar sacs	0.00072	0.00053	73
**Max. wall shear stress (Pa)**	Inhalation	Bronchioles	2.9	5.1	56
		Alveolar sacs	0.09	0.46	20
	Exhalation	Bronchioles	0.7	1.6	43
		Alveolar sacs	0.049	0.24	20

Remarks: 28~42% of difference by experiment [7] for Maximum pressure, and The peak normal wall shear stress was observed in 3.5 Pa at lower airways by simulation data [21], and 53% (maximum) of difference between normal and obstructed airway (aging effect) was found by simulation data [22].

**Table 5:** Quantification of lung airway functional parameters with age and comparison to experimental data.

		50-year-old	80-year-old	Difference (%)
**Max. airway space change by ratio**	Bronchioles	1	0.4	-60
	Alveolar sacs	1	0.5	-50
**Airway resistance (kPa/L/sec)**	Inhalation	0.254	0.273	+7
	Exhalation	0.283	0.277	-2
P–V loop (area) (mL*Pa)		0.0035	0.0059	+41

Remarks: Maximum airway space change between inhalation and exhalation was described by ratio. We assumed maximum airway space change of 50-year-old as reference, it had unit value 1. 30% in bronchiole regions by experimental data [3], and 43% of difference in alveolar regions by experimental data [25] for airway space change. 6% difference of airways resistance by statistical analysis [6], and 1.57%, and 1.45% difference for men and women respectively by statistical analysis [34]. 36% difference of lung compliance by experimental data [7].

The comparison of tissue strain in the bronchiole airways and alveolar sacs between the 50-year-old and the 80-year-old is shown in Figs 6 and 7, respectively. The strain was measured as change in deformation of the model, and the percent strain was computed as maximum principal strain over the inhalation. The strains decreased in the elderly as shown in Fig 6. For bronchiole airways, the longitudinal strain change is the highest (higher strain on 50-year-old, and lower strain on 80-year-old) at G22 during inhalation (t = 0.2 sec) as shown in Fig 6a. Strain difference between the 50-year-old and the 80-year-old is increasing with increasing generation as shown in Fig 6a. Hence, the stiffness difference between the two aged subjects is increasing (higher stiffness for the 50-year-old, more flaccid for the 80-year-old) at G22. This result at G22 also indicates that higher volume change in the elderly leads to reduced compliance.

Stiffness in the airways is defined as resistance to an elastic structure by deformation. The strain is expressed as the ratio of total deformation to the initial dimensions of the structure. The maximum circumferential strain (related to change in diameter of the airways) change in G16 is observed at position 6 during inhalation as shown in Fig 6b. The trends for stress and strain in region 2 are similar to region 1. However, the magnitude of the difference in stress and strain in region 2 between 50-year-old and 80-year-old is less than that in region 1.

**Fig 8:**
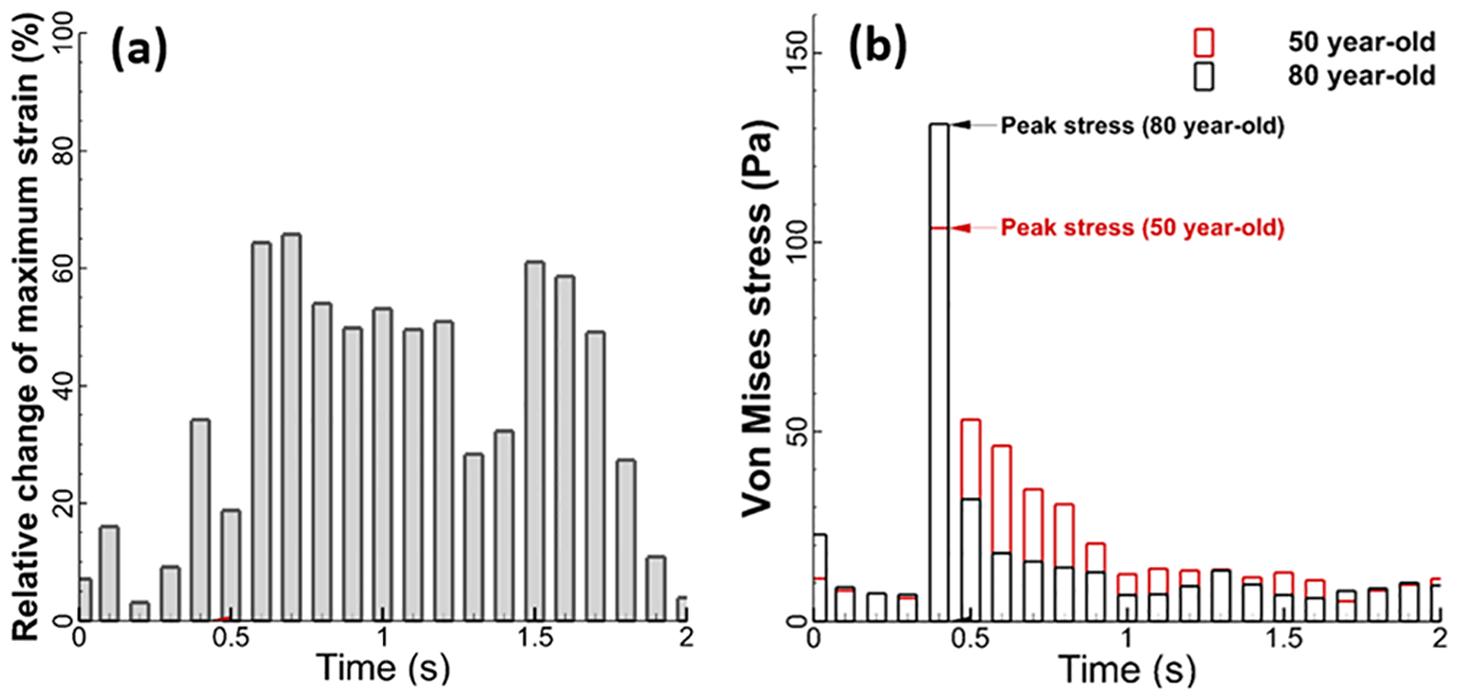
Comparison of mechanical characteristics of airway wall in breathing cycle (inhalation: 0 ~ 0.4 second, exhalation: 0.4 ~ 2.0 second), (a) relative change of maximum strain between 50-year-old and 80-year-old, and (b) Von Mises stress of airway wall (tissue) for 50- and 80- year-old.

The tissue strains in the alveolar sacs for the 50-year-old and the 80-year-old are presented in Fig 7a and 7b. This data was collected from FSI simulations, and each strain along the wall from position 1 to 5 of the alveolar sac was measured. Their values on each position were fitted by spline curve. The longitudinal strain (related to length changes in the airways) results showed the strain was highest at the middle during inhalation (t = 0.2 sec), and also the highest strain change between the 50-year-old and the 80-year-old was observed at position 3 as shown in Fig 7a. Thus, the stiffness difference between the two subjects will be highest at the middle of the alveolar sac. Circumferential strain in the alveolar sac is shown in Fig 7b. The difference in strain between the 50-year-old and the 80-year-old is higher at positions 3 and 5. The relative changes in maximum strain and von-Mises stress for the 50-year-old and 80-year-old during mechanical ventilation are shown in Fig 8. The maximum strain change is observed to be higher during exhalation (t = 0.4 ~ 2.0 sec) than inhalation (t = 0 ~ 0.4 sec) as shown in Fig 8a.

For the von-Mises stress, the 80-year-old had higher values in comparison to the 50-year-old. There is a 25% increase in von-Mises stress for the 80-year-old as compared to the 50-year-old as shown in Fig 8b. For the difference in stress between the 50-year-old and 80-year-old during mechanical ventilation, there is a 50% increase in peak strain in the 80-year-old as compared to the 50-year-old during exhalation. Both the tissue strain and stress results as shown in Figs 6–8 demonstrate that aging affects the lung mechanics parameters.

### Lung function characteristics

**Fig 9:**
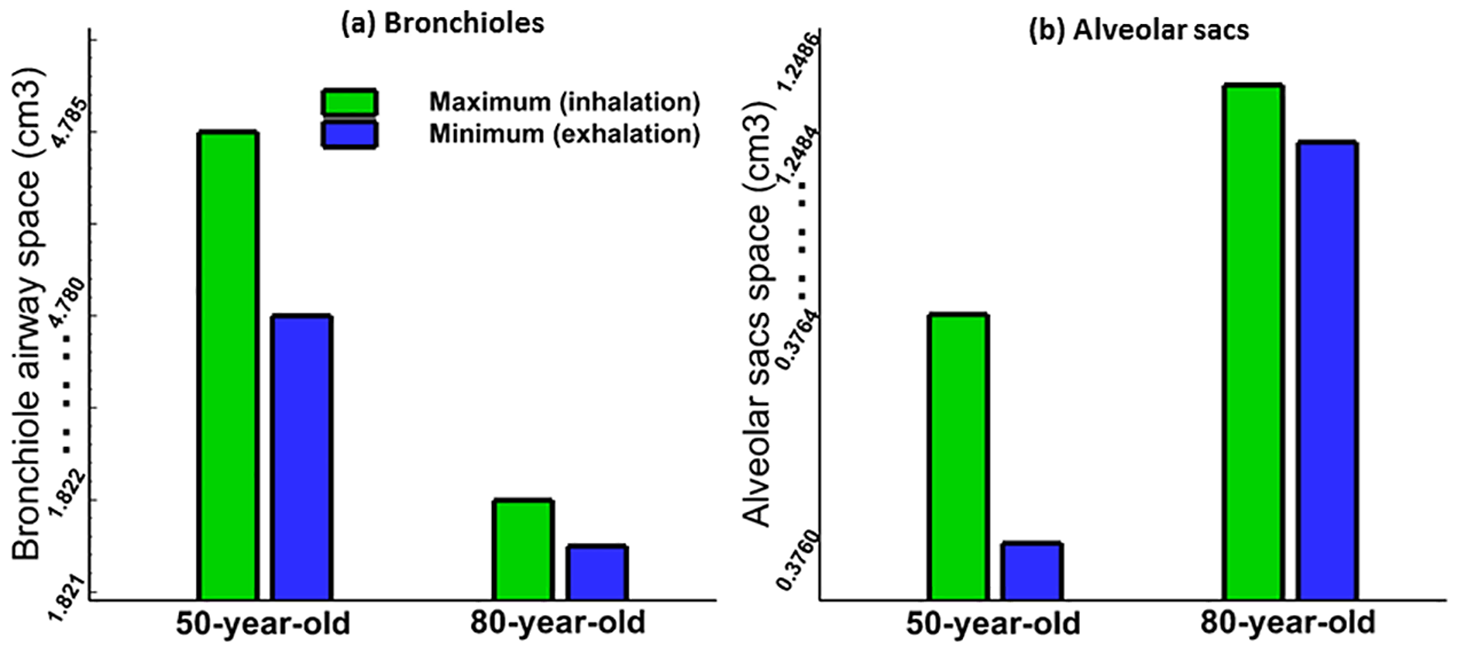
Variation of airway space: (a) airway space change with age at bronchiole region and (b) alveolar sacs.

**Fig 10:**
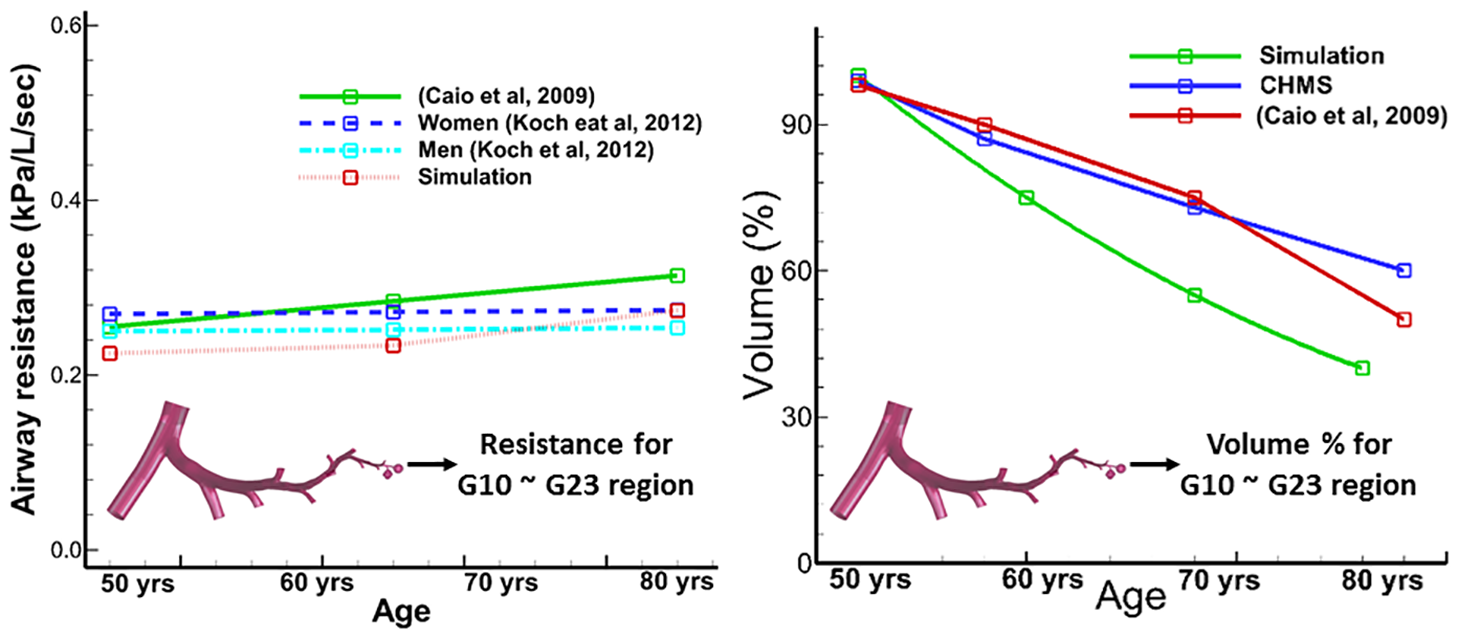
Variation of Airway resistance and air-volumes with age. Airway resistance was compared by Caio’s [6] and Koch’s [48] experiments, and volume also compared with Canadian Health Measures Survey (CHMS) [62] and Caio’s [6] study.

The lung function characteristics represented by airway space and airway resistance parameters were compared for the 50-year-old and 80-year-old during mechanical ventilation and the results are shown in Figs 9 and 10. In this study, the maximum bronchiole airway space was measured during inhalation and exhalation. It can be seen from Fig 9 that the bronchioles airway space decreases with age, by approximately 60% for the 80-year-old in comparison to the 50-year-old. Thus, the 80-year-old bronchiole airway space expanded by less than 60% as compared to the airway space of the 50-year-old. This indicates that higher stiffness of airway wall in a given tidal volume for an 80-year-old led to less expansion of airspace as compared to a 50-year-old. In contrast, the alveolar sacs space varied during inhalation and exhalation, and increased by 50% for the 80-year-old old in comparison to the 50-year-old. Similar trends were observed during inhalation and exhalation for both ages. The results of airway resistance (defined as the pressure divided by the airflow rate) with age are shown in Fig 10. The pressure we used to graph resistance in Fig 10 was maximum pressure. There is only a small difference (about 7% during inhalation, and 3% during exhalation) in airway resistance between the 50-year-old and the 80-year-old. The results for airway volume with age are shown in Fig 10b. The volume percentage was computed as tidal volume over end-expiration volume, and percent airway decreased with age in our simulation.

**Fig 11:**
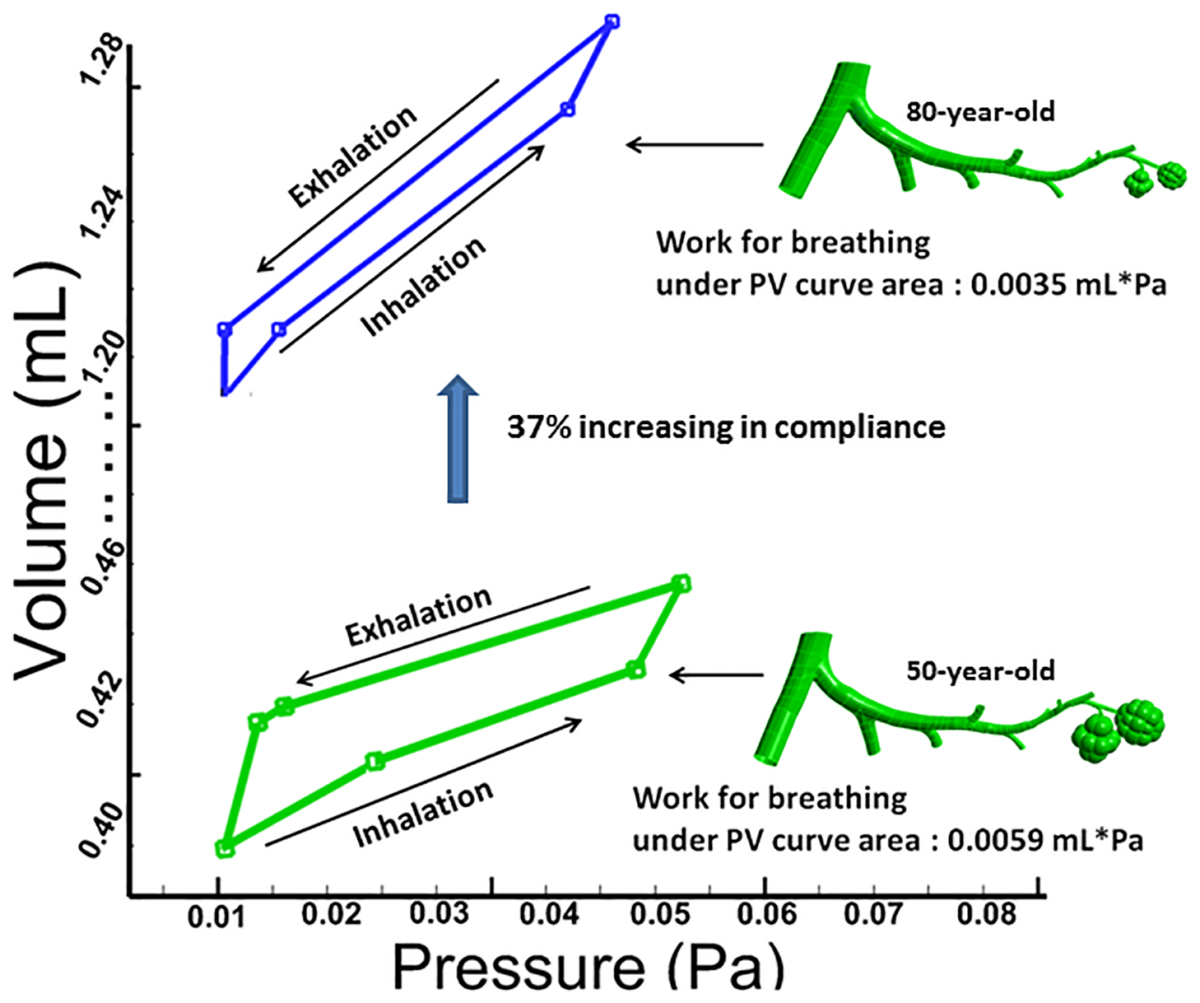
Comparison of relationship with volume-pressure loops for 50-year-old and 80-year-old.

The Pressure-Volume loop for the two cases (50-year-old and 80-year-old) is shown in Fig 11. Pressure and volume were measured over the G10 ~ G23 region and mean values for pressure and volume are presented in Fig 11. It can be seen from Fig 11 that the lung compliance (defined as the difference of volume divided by difference of airway pressure) represented by the slope of the P-V loop diagram, increases with age. This result also indicates that the 80-year-old has to exert more work (about 1.7 times more) than the 50-year-old to take in a normal breath. Overall, there is a 37% increases in lung compliance for the 80-year-old in comparison to the 50-year-old as shown in Fig 11. A summary of these results is presented in Table 5.

## Discussion and conclusion

In this study, computational simulations of human bronchioles model and alveolar sac (generation G10 to G23) model under mechanical ventilation were used to estimate the effects of aging. We hypothesized that aging greatly affects respiratory mechanics under mechanical ventilation. This hypothesis was investigated through analysis of the simulation results of respiratory mechanics parameters (pressure, shear stress, and strain) as well as lung function characteristics (compliance: P- V loop).

It is evident from experimental studies [4, 7, 47] that the compliance of the lung airways increases with age. This observation was quantified through simulations and we found that there was a 37% increase in compliance for the 80-year-old as compared to the 50-year-old, suggesting that extra work would be required to bring in normal volume of air for an 80-year-old. Also, the airway resistance changed very slightly with aging, specifically, about 2–7%, from age 50 to 80, and this result compared well with the experimental data [6, 48]. Normally, when lung volume is controlled, age has no critical impact on airway resistance. The results presented in Fig 10 are in good agreement with existing experimental data for men and women [48] and illustrate that there is minimal change in airway resistance. The flow resistance in addition to the airway resistance is attributed to the airway’s morphology. Due to the decrease in diameter of the small airways morphology in the elderly, the flow resistance change is very small. This result is consistent with experimental measurements [7] and is thus validated [36, 47]. Elastic recoil describes the rebound of lungs after having been stretched by inhalation or rather the ease with which the lung rebounds. Elastic recoil is inversely related to lung compliance. Hence, low lung compliance indicates a stiff lung, and high elastic recoil can be thought of as a thick balloon. This aspect magnifies with age due to larger volumes needed for lung expansion and chest wall compliance and lung function [49]. For the elderly, lung compliance increases (lungs become stiffer) which implies alveolar sacs require relatively more normal gas exchange [48].

Fig 11 depicts the static pressure-volume relationship of the lungs for a 50- and an 80-year-old. The general shape of the curve is not dissimilar, but the position of the curve for elderly subjects shifted to the left (i.e. larger compliance) [7]. This result is consistent with earlier findings [8, 16] that due to rearrangements of elastin fibers and collagen content, certain local regions of the lung may experience high compliance due to changes in tissue properties and respond differently during breathing. The bulk modulus (K) describes uniform inflation and is represented by the reciprocal of specific lung compliance. The rate of the increase in K with age increased with the increase in pressure as reported by Lai-Fook [5]. At each pressure, K increased significantly with age. With increasing bulk modulus, the increased lung volume at each pressure with age was consistent with an increase in lung tissue elastin and a constant collagen content [50]. Thus, there was a tendency of the lung tissue to become more compliant in the elderly. The simulation results of length stiffness and volume changes with age are in good agreement with previous studies [11, 51, 52] and within vivo measurements in humans [4].

Airway strains and stresses were decreased in the elderly (see Figs 6 and 7). The decreased strain and stress rates in the airways (flaccid lung airway) make the elderly more susceptible to lung injury under mechanical ventilation. Lung strain directly reflects changes in lung tissue mechanics and is associated with VILI [53]. In higher respiratory system compliance, strain may decrease (elderly case) and may fall below the safe threshold for VILI [54]. A recent study [55] indicated that lung strain values of approximately 0.27 or less indicate VILI. Additionally, when the strains fall out of the physiological range, excessive lung expansion or alveolar collapse induces VILI [56, 57].

With increasing age, the airway wall becomes more flaccid, and as a result, lung elastic recoil is diminished [58]. At low strain in the 80-year-old, the stress in the bronchioles was tolerated predominately by the elastic fibers in the connective tissue [59]. Collagen in connective tissue, by contrast, was much stiffer and could only bear a small amount of strain before rupturing [60, 61]. Overall, the simulation results indicate that there is a 37% increase in lung compliance, and about 35% change in airway mechanical characteristics for the 80-year-old in comparison to the 50-year-old under mechanical ventilation. Also, these age-related changes (lung compliance and airway mechanical characteristics) in lung physiology increase the severity of lung injury under mechanical ventilation.

The results of pressure-volume loop obtained from simulations demonstrated that there is a 37% increase in lung compliance between the 80-year-old and the 50-year-old. This increase will result in changes in respiratory mechanics, which can be monitored by a clinician to reduce the related lung injury due to mechanical ventilation [62]. Due to a lack of experimental data on aging effects especially in the lower bifurcations and alveolar sacs, the developed computational models and results reported here may be incorporated in experimental studies to quantify the aging effects in human bronchioles and alveolar sacs. These computational results will also help to develop age dependent mechanical ventilation strategies to avoid VILI/ARDS for the elderly in the future.

Limitations of this study include use of limited bronchioles airway models, and a single layer of airway tissue. Due to the complexity of FSI studies, only one layer of the tissue at bronchioles and alveolar sacs was modeled in this study. However, the thickness and material properties of tissue are quite comparable to the reported lung tissue properties [38, 39]. In the future, the lung tissue should be modeled with multiple tissue layers with heterogeneous material properties. Tethering from pleural cavity or diaphragm due to lengthening of the airway bifurcations was not considered in this study and may be addressed in the future. The thickness between the septal wall and the neighboring alveoli was not considered in this study and this aspect of septal thickness will need to be addressed to investigate the effects of mechanical interaction of airways and the surrounding connective tissues.

## Supporting information

S1 File Table of airway model equations for lung geometry in word file. (DOCX)

S2 File Airway cast for lung geometry in stl file. (STL)

S3 File User defined function data for mechanical ventilation in c file. (C)
