# Supplementary material for: Aging effects on airflow dynamics and lung function in human bronchioles
Source: PLoS One. 2017 Aug 28;12(8):e0183654. doi: 10.1371/journal.pone.0183654 (PMC5573216; doi:10.1371/journal.pone.0183654)
Supplement: S1 File — (DOCX) [file pone.0183654.s001.docx]

**Supporting Information**

**Supplementary equation and table I for lung geometry**

The number of elements in each generation *z* is evidently

*n(z)* = $2^{z}$

Diameter and length of the single cylindric elements are given by the pairs of empiric equations fitted to the measurements obtained on case and histologic preparations. Table I lists these equations along with the formulas for the dimension *D(z)*, the length *L(z)*, the overall cross section *A(z)* and the overall volume *V(z)* of the airways in each generation as they could be derived from equations in Table I. This airway model refers to an average-sized adult human lung.

**Table. Airway model equations for lung geometry**

| $D(z)$ | $D_{0}\cdot e^{-0.388z}$ | $if z\leq3$ | $D_{0}=1.8 cm$ |
| --- | --- | --- | --- |
|  | $D'_{0}\cdot e^{-(0.2929-0.00624z)z}$ | $if z>3$ | $D'_{0}=1.3 cm$ |
| $L(z)$ | $L_{0}\cdot e^{-0.92z}$ | $if z\leq3$ | $L_{0}=12 cm$ |
|  | $L'_{0}\cdot e^{-0.17z}$ | $if z>3$ | $L'_{0}=2.5 cm$ |
| $A(z)$ | $A_{0}\cdot e^{-0.083z}$ | $if z\leq3$ | $A_{0}=2.54 cm^{2}$ |
|  | $A'_{0}\cdot e^{(0.17074+0.0125z)z}$ | $if z>3$ | $A'_{0}=1.32 cm^{2}$ |
| $V(z)$ | $V_{0}\cdot e^{-1.003z}$ | $if z\leq3$ | $V_{0}=30.5 cm^{3}$ |
|  | $V'_{0}\cdot e^{(0.0125z-0.0626)z}$ | $if z>3$ | $V'_{0}=3.3 cm^{3}$ |
